# Human Umbilical Cord Blood Cell Transplantation in Neuroregenerative Strategies

**DOI:** 10.3389/fphar.2017.00628

**Published:** 2017-09-08

**Authors:** Luisa R. Galieva, Yana O. Mukhamedshina, Svetlana S. Arkhipova, Albert A. Rizvanov

**Affiliations:** ^1^OpenLab Gene and Cell Technologies, Institute of Fundamental Medicine and Biology, Kazan Federal University Kazan, Russia; ^2^Department of Histology, Cytology and Embryology, Kazan State Medical University Kazan, Russia

**Keywords:** human umbilical cord blood cells, neuroregeneration strategies, neurodegeneration diseases, spinal cord injury, stroke

## Abstract

At present there is no effective treatment of pathologies associated with the death of neurons and glial cells which take place as a result of physical trauma or ischemic lesions of the nervous system. Thus, researchers have high hopes for a treatment based on the use of stem cells (SC), which are potentially able to replace dead cells and synthesize neurotrophic factors and other molecules that stimulate neuroregeneration. We are often faced with ethical issues when selecting a source of SC. In addition to precluding these, human umbilical cord blood (hUCB) presents a number of advantages when compared with other sources of SC. In this review, we consider the key characteristics of hUCB, the results of various studies focused on the treatment of neurodegenerative diseases (Alzheimer's disease, Parkinson's disease, amyotrophic lateral sclerosis), ischemic (stroke) and traumatic injuries of the nervous system and the molecular mechanisms of hUCB-derived mononuclear and stem cells.

## Cell therapy strategies for nervous tissue repair

Until the middle of the last century, it was believed that neural regeneration was impossible. In this regard, neurodegenerative diseases (Alzheimer's disease, Parkinson's disease, amyotrophic lateral sclerosis), ischemic (stroke) and traumatic injuries of the nervous system were considered incurable and only symptomatic treatment was offered. Even today, there is no effective treatment of the above-mentioned diseases. However, scientists and clinicians have high expectations for a treatment based on the use of stem cells (SC), which can potentially replace dead cells and synthesize both neurotrophic factors and molecules that stimulate neuroregeneration. Nowadays, stem cell therapy is seen as a promising method for the treatment of neurological diseases. Unfortunately adverse effects and ethical issues have forced researchers to seek non-fetal sources of SC.

Human umbilical cord blood (hUCB) is widely used as a rich and ethically acceptable source of SC with high regenerative potential. hUCB is easily accessible, can be obtained without risk to the donor and is rarely contaminated with infectious agents, such as cytomegalovirus or Epstein–Barr virus (Rubinstein et al., 1993). hUCB contains a high level of CD34^+^ and CD105^+^ cells (SC markers), which are an indication of high regenerative potential (Verneris and Miller, 2009). Moreover, hUCB is a source of cells characterized by a lower oncogenic potential and longer telomeres (McGuckin and Forraz, 2008). It should be noted that hUCB cells have a lower probability of immune rejection, since they are more tolerant to differences in human leukocyte antigen (HLA) (Danby and Rocha, 2014).

## Human umbilical cord blood cells types and potential

hUCB contains several types of stem and progenitor cells. Hematopoietic stem cells (HSCs), mesenchymal stem cells (MSCs), endothelial progenitor cells (EPCs), and unrestricted somatic stem cells (USSCs) are the most frequently used to stimulate neuroregeneration (van Rood et al., 2009; Jiao et al., 2012). Today, most of the preclinical trials assessing the role of hUCB cells in processes stimulating neuroregeneration exploit its mononuclear fraction (hUCB-MCs) (Pimentel-Coelho et al., 2012).

### Hematopoietic stem cells

These cells were the first isolated from hUCB. The original mention of hUCB as a potential source of HSCs was published in 1972 (Ende and Shende, 1972). But it was only in 1986 that HSCs could be successfully isolated from hUCB by Broxmeyer (Broxmeyer et al., 1989). The first transplantation of HSCs isolated from hUCB was performed in 1988 in a 5 year old with Fanconi anemia (Gluckman et al., 1989). The surface phenotype of HSCs is poorly determined. But the presence of CD34 and the absence of AC133, CD38, and HLA-DR determine the ability of a HSC population to restore hematopoiesis (Yin et al., 1997).

For 30 years, hUCB has caught the attention as an important source of HSCs, owing to the fact that hUCB has several advantages over bone marrow. hUCB HSCs show a higher immune tolerance regarding HLA compatibility between donor and recipient and there is less probability of graft vs. host disease (GvHD) due to the immaturity of the neonatal immune system (Gluckman et al., 1997; Goldstein et al., 2007; Danby and Rocha, 2014). At the same time, hUCB transplantation can be associated with a longer engraftment, poor immune reconstitution and higher rates of infection compared to conventional sources of HSCs (Danby and Rocha, 2014). It is can be due to the quantitative and qualitative differences in the cell populations in hUCB grafts (Rocha and Broxmeyer, 2010). It has also been reported that hUCB contains an equal or larger quantity of HSCs compared with patient bone marrow (Rogers and Casper, 2004).

It is worth noting that transplantation of HSCs is applied primarily in the treatment of hematological and oncological diseases, and also autoimmune and cardiovascular diseases. However, one research team has shown that hematopoietic progenitor cells differentiate into microglia when transplanted into an intact spinal cord (Eglitis and Mezey, 1997). Also, it has been reported that HSCs can be differentiated *in vitro* to astrocytes and oligodendrocytes using retinoic acid (Jang et al., 2004).

### Mesenchymal stem cells

These cells can be isolated from hUCB, as well as from bone marrow, placenta, adipose tissue, dental pulp and parenchymal organs (Erices et al., 2000; Campagnoli et al., 2001; Scherjon et al., 2004; Wang et al., 2004). More than 95% of MSCs express the antigens CD73, CD90, and CD105 on the cell surface, but do not express CD45, CD34, and CD14 (Gluckman et al., 1997). They are characterized by a high proliferative activity and a bias toward differentiating *in vitro* into osteoblasts, chondroblasts, adipocytes and stromal cells, which form the hematopoietic microenvironment (Kim et al., 2013). However, the MSCs differentiation potential in the neurogenic direction with possible functional consistency has remained a controversial question. Today, bone marrow is considered the main source of MSCs. But the extraction of bone marrow is still an invasive and very painful procedure. In addition, a significant disadvantage is that the number and the differentiation potential of MSCs, their proliferative activity and life span decrease with age (Stenderup et al., 2003). To date, the adipose-derived MSCs are becoming more popular and are good alternative to BM-MSCs, they are not inferior to the latter and their harvesting is not associated with to the above mentioned problems. At the same time, mechanisms of their effect on neuroregeneration are not clearly understood. Nevertheless, adipose-derived MSCs have strong translation potential for clinical applications.

Thus hUCB is an alternative source of MSCs (hUCB MSCs). It should be noted that MSCs isolated from different sources have common characteristics: typical morphology; growth pattern in culture; ability to differentiate under the influence of specific stimulants into osteogenic, adipogenic, and chondrogenic precursors; support of hematopoiesis *in vitro*; expression of common surface markers and identical expression profile of most genes. However, there are some differences among MSCs obtained from different tissues and organs. In this regard, it is worth noting that neonatal cells have more advantages owing to their immaturity. In particular, hUCB MSCs express genes associated with the three germinal layers (Kang et al., 2013). It has been established that hUCB MSCs are more committed to angiogenesis, whereas bone marrow MSCs are more committed to osteogenesis. hUCB MSCs have a higher proliferative activity and a lower expression of both CD106 and HLA-ABC, this being an indication of a lower alloreactivity of these cells.

Mesenchymal stem cells (MSCs) are actively used as therapeutic agents in the treatment of various human neurodegenerative diseases (Vercelli et al., 2008; Glavaski-Joksimovic and Bohn, 2013; Hsieh et al., 2013; Kim et al., 2015). However, widespread therapeutic use of MSCs is hindered by limitations in their optimal preparation, dosing and delivery. So, human MSC levels in tissue varied widely according to tissue site and harvest method. Previously, Vangsness et al. shown that yields from adipose tissue, bone marrow and umbilical cord tissue ranged from 4,737 to 1,550,000 cells/mL, 1–30 to 317,400 cells/mL, and 10,000 cells/mL to 4,700,000 cells/cm of tissues, respectively (Vangsness et al., 2015). The main obstacle to getting MSCs into the CNS is the blood–brain barrier (Aleynik et al., 2014). Several studies have reported the possibility of differentiation of hUCB MSCs *in vitro* into neural cells (Fu et al., 2004; Karahuseyinoglu et al., 2007). Chua et al. have used hUCB-derived multipotent stem cells. These cells have properties similar to those of multipotential mesenchymal cells found in the bone marrow (Chua et al., 2010).

### Endothelial progenitor cells

These and HSCs are derived from a common hemangioblast precursor. EPCs are also present in peripheral blood, but their concentration in hUCB is significantly higher. The expression of CD34, vascular endothelial growth factor (VEGF) and Tie-2 (one of the angiopoietin receptors) is characteristic of EPCs. Cultured EPCs differentiate into network forming endothelial cells. Their transplantation induces neovascularization in mouse models of stroke (Murohara, 2001; Taguchi et al., 2004). hUCB EPCs promote greater angiogenesis *in vivo* compared to EPCs derived from peripheral blood. In addition, the co-transplantation of hUCB EPCs and pericyte precursors leads to the formation of long and functioning blood vessels, which provides an attractive platform for tissue engineering (Au et al., 2008).

### Unrestricted somatic stem cells

These cells express CD13, CD29, CD44, CD90, CD49e, and CD105 (Kögler et al., 2004). Their distinguishing feature is the ability to differentiate in the ectodermal, mesodermal and endodermal directions (Danby and Rocha, 2014). It has been shown that USSCs can differentiate into hematopoietic cells, osteoblasts, chondroblasts, adipocytes, neurons and astrocytes both *in vivo* and *in vitro* (Zaehres et al., 2010; Bakhshandeh et al., 2011).

These cells have therapeutic potential in myocardial infarction. They also reduce the likelihood of GvHD (Handschel et al., 2010; Langenbach et al., 2011). USSCs, albeit a small population in hUCB compared to HSCs, reproduce rapidly, even in a serum-free medium, providing sufficient cell quantity for transplantation (Zaibak et al., 2009). Unlike embryonic stem cells, none of the main stem cell markers (Oct4, Sox2, and Nanog) are appreciably expressed in USSCs (Santourlidis et al., 2011). Although the mechanisms underlying USSC multipotency are still unexplored, these cells act as a promising source for cell transplantation.

### hUCB-MCs

At present, most of the preclinical trials assessing the part played by hUCB cells in processes stimulating neuroregeneration work with hUCB-MCs, which can be isolated by density gradient and survive long term preservation (Pimentel-Coelho et al., 2012).

In addition to stem and progenitor cells, there are other cell types in the mononuclear fraction of hUCB, namely regulatory T cells, natural killer (NK) cells, T lymphocytes and dendritic cells. There are two different subpopulations of mononuclear hUCB cells: adherent and floating. The researchers observed more glial antigens expressed in the adherent population and more neuronal antigens in the floating population (Chen et al., 2005). So, these cells can have a positive effect on the course of neurodegenerative diseases.

Immunosuppressive subpopulation CD4^+^ T cells and regulatory T cells (Tregs) play an important role in suppressing the immune response, in the tolerance to own antigens, and prevention of autoimmune diseases. Given the strong anti-inflammatory activity of Tregs, it is believed that these cells exert a significant neuroprotective effect after stroke through the release of IL10 (Dalous et al., 2012). Also, the lymphocytes in hUCB express cytokines, such as interleukin IL2, IL6, and IL7, tumor necrosis factor α (TNF-α) and interferon γ (IFN-γ) and their receptors, to a lesser extent than adult blood cells (Zola et al., 1995; Gluckman and Rocha, 2005). Lymphoid dendritic cells that are present in hUCB induce anti-inflammatory T-helper cells, which along with the naive T-cells may down-regulate immune responses (Arpinati et al., 2000; Willing et al., 2007). NK cells also appear as a subpopulation of circulating lymphocytes. It has been shown that hUCB-NK cells contribute to a lower incidence of GvHD (Schira et al., 2012).

It is known that after allotransplantation there is a risk of GvHD occurrence. But, because of its unique cell populations, hUCB acts as a safe source of stem and progenitor cells that is less likely to cause GvHD, hence reducing the problems of donor search time and preparation for treatment. It is also worth noting that recently in many countries more and more banks (including banks for personalized storage) of hUCB are being established that in the long term would allow to conduct autologous transplantations in which there are no immune complications. In addition, hUCB cells have many properties which allow them to overcome the hurdles of neuroregeneration, which determines some success in the treatment of neurological diseases. Some of these achievements will be discussed below.

## Method of hUCB cells delivery

The method of hUCB cells delivery is of great importance to the clinician. Direct delivery is desirable in that hUCB cells are administered at the site of interest. However, damage from the injection has to be minimal and/or mitigated (Awad et al., 2015). hUCB cells exhibit tropism for sites of tissue damage, which allows the use of less invasive methods of delivery, for example, intravenous and intrathecal administrations. hUCB cells are able to pass through blood–brain barrier, however systemic delivery of these cells leads to insufficient number of cells at the sites of neurodegeneration due to primary deposition of transplanted cells in lungs, spleen and liver. This is why there is still a question about preferred route of hUCB administration—more damaging direct injection at the site of neurodegeneration of large number of cells or systemic delivery with low number of cell actually migrates to the site of neurodegeneration. Table 1 provides a summary list of preclinical trials using hUCB cells with different routes of delivery and their outcomes.

**Table 1:** Preclinical trials using hUCB cells.

**Cell type**	**Condition**	**Method of delivery**	**Outcome**	**References**
**SPINAL CORD INJURY (SCI)**				
hUCB-derived multipotent stem cells	clip-compression model of SCI at T6–T7 (rats)	intraspinal transplantation, immediately after SCI	demonstrated the possibility of expression by hUCB cells NF-200 (neural marker) and CNPas (oligodendrocyte marker) and anti-apoptotic effects	Chua et al., 2010
hUCB-MCs	contusion model of SCI at T10 (rats)	intraspinal transplantation, through 1 week after SCI	anti-apoptotic effects	Dasari et al., 2008
	clip-compression model of SCI at T8-T9 (rats)	intravenous transplantation, through 1 or 5 days after SCI	ameliorate some of the behavioral effects of SCI	Saporta et al., 2003
	clip-compression model of SCI at T8-T9 (rats)	intravenous transplantation, immediately after SCI	improvement of motor functions	Chen et al., 2008
	contusion model of SCI at T10 (rats)	intraspinal transplantation, through 1 week after SCI	anti-inflammatory effect	Veeravalli et al., 2009
hUCB-MSCs	balloon-compression model of SCI at L1 (dogs)	intraspinal transplantation, through 1 week after SCI	anti-inflammatory effect, increase in number of regenerating nerves	Ryu et al., 2012
hUCB-USSCs	hemisection model of SCI at Th8 (rats)	intraspinal transplantation, immediately after SCI	significantly increase in number of regenerating axons within the lesion area	Schira et al., 2012
**ALZHEIMER'S DISEASE (AD)**				
hUCB-MCs	PSAPP and Tg2576 (mice)	intravenous transplantation	reduction of cerebral Aβ-peptide, anti-inflammatory effect	Nikolic et al., 2008
	APP/PS1 (mice)	retro-orbitally transplantation	positive clinical effects	Petukhova et al., 2015
hUCB-MSCs	APP/PS1 (mice)	intravenous transplantation	improvement of spatial learning and memory	Ende et al., 2000a
**AMYOTROPHIC LATERAL SCLEROSIS (ALS)**				
hUCB-MCs	SOD1-G93A (mice)	retro-orbitally transplantation	improvement of motor functions and neuromuscular transmission, decrease in motor neurons death	Souayah et al., 2012
	SOD1-G93A (mice)	intravenous transplantation	slowing of disease progression, increase of lifespan, enhance motor neuron survival, modulate gliosis, reduce activation of microglia and astrocytes	Garbuzova-Davis et al., 2012
	SOD1-G93A (mice)	intravenous and nretro-ocular transplantations	significant increase of mice lifespan	Chen and Ende, 1999; Ende et al., 2000b
	SOD1-G93A (mice)	intracerebro-ventricular transplantation	beneficial effect of motor neuron degeneration	Bigini et al., 2011
hUCB cells	SOD1-G93A (mice)	intravenous transplantation	cell migration predominantly in area of neurodegeneration	Garbuzova-Davis et al., 2003
hUCB-HSCs	SOD1-G93A (mice)	intraspinal transplantation	improvement of motor functions, increase of mice lifespan	Knippenberg et al., 2011
**PARKINSON'S DISEASE (PD)**				
hUCB-MSCs	6-OHDA lesion (mice)	transplantation in the right substantia nigra	improvement of motor functions, demonstrated the possibility of expression by hUCB-MSCs nestin, NeuN, NGF, TH, MAP2, NF, β-tubulin III	Kang et al., 2013
hUCB-MCs	BbCBACa-AW-J/A-K cnj6 <wv> (mice)	intravenous transplantation of megadoses hUBC-MCs	significant delay the onset of symptoms and death of mice	Ende and Chen, 2002
**STROKE**				
hUCB-MSCs	reperfusion model (rats)	intracerebral transplantation	the formation of new blood vessels, hUBC-MSCs migrate to the ischemic area and express MAP-2, GFAP and Neu-N	Ding et al., 2007
hUCB-USSCs	MCAO (rats)	intracerebral transplantation	strengthening of hUCB-USSCs migration by HGF, expressing by apoptotic neurons in ischemic area	Trapp et al., 2008
hUCB cells	MCAO (rats)	intravenous transplantation	anti-inflammatory effect	Vendrame et al., 2004
	MCAO (rats)	intravenous transplantation	upregulation the expression of white-matter-associated proteins after ischemia	Rowe et al., 2010
	MCAO (rats)	intravenous transplantation	protective effect on oligodendroglia	Rowe et al., 2012
	MCAO (rats)	intravenous transplantation	improvement of motor functions	Chen et al., 2001
	MCAO (rats)	intravenous and intraparenchymal transplantations	reduction in lesion volume and mitigation of symptoms, intraparenchymal transplantation leads to axonal sprouting	Xiao et al., 2005
hUCB-HSCs	MCAO (rats)	intravenous and intrastriatal transplantations	improvement of neurological function equally for both variants of delivery	Willing et al., 2003
hUCB-MCs	MCAO (rats)	intravenous transplantation	reduction in stroke-induced infiltration of microglia/macrophages and B cells, anti-inflammatory effect	Vendrame et al., 2005

### Current perspectives in the treatment of SCI

Human umbilical cord blood (hUCB) can promote functional recovery after SCI through several mechanisms. It has been shown that hUCB cells secrete anti-inflammatory cytokines and growth factors. In particular, it has been proven that systemic injections of hUCB as a treatment for SCI improves the restoration of hind limb function by stimulating the synthesis of IL10, glial cell line-derived neurotrophic factor (GDNF) and vascular endothelial growth factor (VEGF) (Chen et al., 2008). Moreover, the secretion of TIMP-1 and TIMP-2, both inhibiting matrix metalloproteinases involved in inflammatory processes, has been observed following hUCB-derived multipotent stem cells transplantation into the site of SCI (Chua et al., 2010). It is also worth noting that transplantation of hUCB-MCs into the damaged site reduces the amount of Fas proteins, caspases and other proteins associated with apoptosis (Dasari et al., 2008). It has been found that hUCB-MCs transplantation also significantly reduces the activity of tissue plasminogen activator (tPA), which is synthesized after spinal cord injury and contributes to both tissue damage and loss of limb motor function (Veeravalli et al., 2009). It has been shown that the intravenous administration of hUCB MSCs during the acute phase between 1 and 5 days after SCI may alleviate some of the behavioral effects of SCI. In addition, the researchers came to the conclusion that the intravenous introduction of hUCB cells has distinct advantages over the more conventional direct transplantation of cells into the injury site (Saporta et al., 2003).

Stored myelin sheaths around the axons in the spinal cord damaged areas were observed following intraspinal transplantation of hUCB USSCs. However, hUCB USSCs did not differentiate into neurons, astrocytes, oligodendrocytes or Schwann cells after transplantation into the acutely injured spinal cord (Schira et al., 2012).

As it was shown in Lee et al. (2011), hUCB MSCs *in vivo* may also enhance the remyelination by peripheral myelin sheaths. It has been reported that the transplantation of extracellular matrix Matrigel and hUCB MSCs in dog models of SCI is associated with a large number of regenerating nerves, neuroprotection and less inflammation, exceeding the results of transplantation of MSCs isolated from other sources (Ryu et al., 2012). In addition, it was shown in Dasari et al. (2008) that trans-differentiation into neurons and oligodendrocytes, both *in vitro* and *in vivo*, is possible following transplantation of hUCB cells into an injured spinal cord.

Given the promising results of the preclinical studies described above, the Food and Drug Administration (FDA) approved a clinical trial using hUCB cells for treatment of SCI (Table 2). In a phase I/II trial, 28 patients with chronic complete SCI were monitored after hUCB-MC transplant therapy (Zhu et al., 2016). In this Hong Kong study, 8 patients with an average post-injury period of 13 years were treated. They received an injection of either 1.6 million (4 patients) or 3.2 million (4 patients) hUCB-MCs into dorsal entry zones above and below the injury site. Motor skills, the walking index of SCI (WISCI) and spinal cord independence measure (SCIM) scores in these patients did not change in the 12 months after transplantation. However, two participants had fiber bundles growing across the injury site. In another study, this time in Kunming, 20 patients with an average post-injury period of 7 years were divided into 5 groups of 4 patients, according to the received dose of hUCB-MCs and depending on whether methylprednisolone or oral lithium carbonate was administered (ClinicalTrials.gov Identifier: NCT01046786, NCT01354483). An important detail is that each patient received 3–6 months of intensive locomotor training. The study showed that hUCB-MCs transplants plus locomotor training improved WISCI and SCIM (Zhu et al., 2016). The researchers noted that the intensive training of the musculoskeletal system is essential for the restoration of motor activity, while transplantation of hUCB-MCs in combination with intensive training of the musculoskeletal system may lead to a significant improvement in the functions of the musculoskeletal system, the bowel and the bladder.

**Table 2:** Clinical trials using hUCB cells.

**Cell type**	**Condition**	**Method of delivery**	**Outcome**	**References**
**SPINAL CORD INJURY (SCI)**				
hUCB-MCs	chronic complete SCI	transplantation into dorsal entry zones above and below the injury site	two participants had fiber bundles growing across the injury site	Zhu et al. (2016)
		intraspinal transplantation	hUCB-MCs transplants plus locomotor training improved WISCI and SCIM	Zhu et al. (2016) ClinicalTrials. Identifier: NCT01046786NCT01354483
	acute and subacute SCI	intraspinal transplantation	No Study Results Posted	ClinicalTrials. Identifier: NCT01471613
hUCB-MSCs	chronic SCI	intrathecal transplantation	No Study Results Posted	ClinicalTrials. Identifier: NCT01873547
	subacute and chronic SCI	intrathecal transplantation	No Study Results Posted. This study is currently recruiting participants.	ClinicalTrials. Identifier: NCT02481440
**NEURODEGENERATIVE DISEASES**				
hUCB-MSCs	dementia of Alzheimer's type	intraventricular administrations	No Study Results Posted. Apparently, the researchers were able to determine the optimal dose of hUCB MSCs and the best method of their transplantation to patients.	ClinicalTrials. Identifier: NCT01297218 NCT02054208
	Alzheimer's Disease	intravenous transplantation	No Study Results Posted.	ClinicalTrials. Identifier: NCT01547689
	Alzheimer's Disease	intravenous transplantation	No Study Results Posted. This study is not yet open for participant recruitment.	ClinicalTrials. Identifier: NCT02672306
	Amyotrophic Lateral Sclerosis	intrathecal transplantation	No Study Results Posted.	ClinicalTrials. Identifier: NCT01494480
hUCB	Amyotrophic Lateral Sclerosis, Parkinson's Disease	it is not known	No Study Results Posted.	ClinicalTrials. Identifier: NCT02236065
**STROKE**				
hUCB-MCs	chronic ischemic stroke	transplantation into brain tissue adjacent to the infracted site	No Study Results Posted. The study is still recruiting participants.	ClinicalTrials. Identifiers: NCT02433509
	acute ischemic stroke	intravenous transplantation	No Study Results Posted. This study is currently recruiting participants.	ClinicalTrials. Identifier: NCT01673932
CD34+ stem cells obtained from hUCB	chronic ischemic stroke	intercerebral implantation	No Study Results Posted.	ClinicalTrials. Identifiers: NCT01438593
hUCB	stroke	it is not known	No Study Results Posted.	ClinicalTrials. Identifiers: NCT01884155
	perinatal arterial ischemic stroke	intravenous transplantation	No Study Results Posted.	ClinicalTrials. Identifier: NCT02460484
	ischemic stroke	intravenous transplantation	No Study Results Posted.	ClinicalTrials. Identifiers: NCT02397018
	ischemic stroke	intravenous transplantation	No Study Results Posted.	ClinicalTrials. Identifier: NCT03004976

In China, a phase I/II trial, which started in September 2011 and concluded in January 2014 (ClinicalTrials.gov Identifier: NCT01471613), investigated the safety and efficacy of oral lithium, intraspinal hUCB-MC transplant, and their combination in the treatment of acute and subacute spinal cord injury. Additional clinical studies have been suggested the success of which would determine the further development of therapy using hUCB-MCs.

## Current perspectives in the treatment of neurodegenerative diseases

### Alzheimer's disease (AD)

On the basis of genetic, biochemical and postmortem studies, it has been established that amyloid beta (Aβ) peptide plays a key role in the pathogenesis of AD (Selkoe, 2001). Aβ-peptide exerts pro-inflammatory effect and causes neurodegenerative changes in the brain. The oligomeric forms of this peptide are neurotoxic (Malinin et al., 2005). It has been shown that intravenous administration of hUCB-MCs to transgenic mice with AD (PSAPP and Tg2576 mice) results in a decrease in cerebral Aβ peptide and a reduction of pro-inflammatory responses in the brain and periphery. Intravenous infusion of hUCB mononuclear fraction to transgenic mice results in reduced levels of both soluble and insoluble Aβ peptide and an increase of this peptide in blood plasma. The researchers confirmed that following the introduction of hUCB the Aβ peptide is excreted from the brain tissue through the blood–brain barrier. The reduction of brain inflammation including CD40^+^ activated microglia and GFAP^+^ activated astrocytes occurs in the same way (Nikolic et al., 2008).

It has also been verified that retro-orbital injections of hUCB-MCs exert positive clinical effects in APP/PS1 mice, improving spatial memory, reducing anxiety and nonspecific excitability and increasing the efficiency of exploratory behavior (Petukhova et al., 2014). Moreover, the transplanted cells were detected in the cortex and hippocampus even as late as 3 months after transplantation.

Recently, two phase I/II clinical trials were performed in Korea and China with the purpose of evaluating the safety and efficacy of the use of hUCB MSCs in patients with AD. In Korea, the trial started in February 2011, and was completed in December 2011 (ClinicalTrials.gov Identifier: NCT01297218). This study evaluated the safety and the tolerability of NEUROSTEM®-AD (hUCB MSCs) and assessed the maximum tolerated dose (MTD). Apparently, the researchers were able to determine the optimal dose of hUCB MSCs and the best method of their transplantation to patients. Consequently, this team of researchers started a new clinical trial in February 2014 to investigate the safety, dose limiting toxicity (DLT), and explore the efficacy of three repeated intraventricular administrations of NEUROSTEM® vs. placebo via an Ommaya reservoir at 4 week intervals in patients with AD (ClinicalTrials.gov Identifier: NCT02054208). In March 2012 in China, a phase I/II trial began, which is currently ongoing, but not recruiting new participants (ClinicalTrials.gov Identifier: NCT01547689). The aim of this study is to determine the safety, tolerability and the efficacy of hUCB MSCs after intravenous injection (8 infusions once every 2 weeks in the first month of each quarter) in patients with AD. Another Chinese trial, which started in May 2016, will evaluate the safety and efficacy of hUCB MSCs in patients with AD after 8 intravenous infusions once every 2 weeks, vs. placebo (normal saline) (ClinicalTrials.gov Identifier: NCT02672306). It is hoped that the clinical investigations described above will make it possible to either find an effective variant of cell therapy using hUCB cells in patients with AD or reveal a direction for further studies.

### Amyotrophic lateral sclerosis (ALS)

ALS belongs to the group of neurodegenerative diseases with progressive loss of brain and spinal cord motor neurons. Results indicating that repeated intravenous administration of hUCB-MCs in mouse models of ALS protect primarily motor neurons against inflammatory processes at the onset of symptoms supports the use of hUCB in the treatment of this pathology (Garbuzova-Davis et al., 2012). Moreover, various methods of transplantation (intra-spinal, intra-cerebroventricular) of hUCB cells and hUCB-derived cells to transgenic mice (SOD 1 G93A) have shown the possibility of prolonging survival, reducing the death of motor neurons and activating microglia and astrocytes. At the same time, it was found that the therapeutic effect depended upon the number of transplanted cells (Chen and Ende, 1999; Ende et al., 2000b; Willing et al., 2007; Bigini et al., 2011; Garbuzova-Davis et al., 2012). Other studies have confirmed that the transplantation of hUCB cells and hUCB-derived cells at an early stage of ALS may improve the motor function and neuromuscular transmission, reduce the loss of motor neurons and increase the survival rate of transgenic mice (Knippenberg et al., 2011; Souayah et al., 2012).

Garbuzova-Davis et al. determined the optimal dose of hUCB cells which increased the survival rate of mice and minimized proinflammatory cytokines in the brain and spinal cord (Garbuzova-Davis et al., 2008). Previously, the same team of researchers had shown that intravenously administered hUCB cells migrated predominantly to the neurodegeneration site, although these cells were also found in other organs (Garbuzova-Davis et al., 2003). It was established that the transplanted hUCB cells expressed neural markers, such as nestin, III β-Tubulin (TuJ1) and glial fibrillary acidic protein (GFAP).

These studies demonstrate the possibility of clinical trials, but scientific reports on this use are still limited. Meanwhile, the second phase of another Chinese clinical trial which started in March 2012 on the use of hUCB MSCs in ALS (ClinicalTrials.gov Identifier: NCT01494480) is currently ongoing. These researchers believe that intrathecal injection of hUCB MSCs can secrete trophic factors maintaining motor neuron function. They have designed a phase I/II clinical trial to check the feasibility of this approach in humans. In August 2014 a Korean pilot study of a combination therapy of allogeneic hUCB and granulocyte-colony stimulating factor (GCSF) for patients with brain injury or neurodegenerative disorder (ALS, PD) began, but recruitment status and results are unknown (ClinicalTrials.gov Identifier: NCT02236065).

### Parkinson's disease (PD)

This is a degenerative disease of the extrapyramidal system associated with progressive destruction and death of neurons that produce the neurotransmitter dopamine. According to recent studies, hUCB MSCs exert positive effects in animal models of PD (Kim et al., 2013). In a comparative study of hUCB MSCs vs. BM MSCs, it was found that hUCB MSCs had a greater potential to differentiate into neuron-like cells *in vitro*. Moreover, the transplantation of hUCB MSCs in mouse models of PD has led to behavioral recovery in most animals. These findings support the potential use of hUCB MSCs in the development of viable therapeutic strategies for the treatment of PD (Kang et al., 2013).

Ende et al. found that immature hUBC cells are similar in their properties to embryonic stem cells. Intravenous injections of mega doses of hUBC mononuclear fraction in animal models of PD have prolonged life (Ende and Chen, 2002). Using the same method, a team of scientists has succeeded in delaying the symptoms and prolonging life in animal models of ALS (Chen and Ende, 1999), Huntington's disease (Ende and Chen, 2000), and AD (Ende et al., 2000a). Even though the positive results of hUCB cells transplantation for the treatment of PD have been confirmed in preclinical studies, it has become evident that the available information is not yet sufficient to translate this experience into clinical practice. The only pilot study of combination therapy of allogeneic hUCB and GCSF known to date is not sufficient to draw any conclusion because of the lack of information regarding its results (ClinicalTrials.gov Identifier: NCT02236065).

## Current perspectives in the treatment of stroke

Stroke is caused by impaired blood flow to the brain resulting in damage to and death of nerve cells. The safety and efficacy of intra-cerebral transplantation of hUCB MSCs in animal models of ischemic brain damage have already been proven (Ding et al., 2007; Trapp et al., 2008). It has also been shown that hUCB MSCs migrate to the ischemic site and differentiate into neurons and glial cells. Furthermore, transplantation of hUCB MSCs enables the formation of new blood vessels, thereby increasing blood flow in the ischemic region (Ding et al., 2007).

It has been shown that hepatocyte growth factor (HGF) enhances the migration of transplanted hUCB USSCs. This factor is secreted by apoptotic neurons in the ischemia site, while necrotic neurons, in contrast, do not secrete HGF and are not able to trigger the migration of USSCs (Trapp et al., 2008).

It has also been shown that both intravenous and intrastriatal administration of hUCB cells equally improve neurological functions in animals after stroke (Chen et al., 2001; Willing et al., 2003). It was also discovered that transplanted hUCB cells transdifferentiate into neurons, astrocytes and endothelial cells. In addition, intravenous and intrastriatal transplantations of hUCB-HSCs improve neurological functions equally (Willing et al., 2003). In a rat model of middle cerebral artery occlusion (MCAO) intravenous infusion of non-hematopoietic hUCB cells reduced the damaged area and alleviated the symptoms. Furthermore, the direct transplantation of these cells lead to axonal sprouting. The regenerative effects of these cells suggest that they may be useful in the treatment of ischemic brain injury (Xiao et al., 2005). hUCB cells have been shown to oppose the pro-inflammatory T helper cell type 1 (Th1) response, as demonstrated in an animal model of stroke where hUCB cell infusion promoted a strong anti-inflammatory T helper 2 (Th2) response (Vendrame et al., 2004).

Oligodendrocytes are particularly sensitive in cases of ischemia due to the energy and iron required for the synthesis and maintenance of myelin (Pantoni et al., 1996; Lyons and Kettenmann, 1998). hUCB soluble factors protect and enhance the expression of proliferative, myelin-associated and antioxidant genes in a culture of oligodendrocytes during oxygen and glucose deprivation (Rowe et al., 2010). It has been confirmed *in vivo* that intravenous administration of hUCB cells reduces infarct size and preserves white matter integrity. This effect is due to the fact that systemic infusion of hUCB-MCs enhances Akt phosphorylation and Prdx4 protein expression in white matter after ischemia, which in turn leads to increased cell survival and protection of oligodendroglia (Rowe et al., 2012).

Human umbilical cord blood (hUCB) cell based treatment changes the molecular and cellular processes initiated by cerebral ischemia. Transplantation of hUCB-MCs decreases stroke-induced infiltration of CD45^+^/CD11b^+^ (microglia/macrophages) and CD45^+^/B220^+^ cells (B cells). hUCB-MC-based therapy also reduces the concentration of pro-inflammatory cytokines in the tissue (in particular, TNF-α) (Vendrame et al., 2005). Thus, hUCB cell-based therapy provides anti-inflammatory and neuroprotective effects, improving the clinical outcome after stroke.

Many cell-based clinical studies for stroke are underway using hUCB cells. Most of them are in phase I, aiming to demonstrate the safety and feasibility of hUCB cell transplantation. The first clinical study on the use of hUCB cells for the treatment of stroke was submitted for registration with FDA to begin in September 2012 in USA (ClinicalTrials.gov Identifier: NCT01700166). The purpose of this study was to determine the safety and efficacy of autologous hUCB-derived stem cell injections in children with perinatal arterial ischemic stroke. However, this study was witdrawn prior to enrollment. A month later, in October 2012, the FDA approved clinical study using hUCB cells to treat stroke in China (ClinicalTrials.gov Identifier: NCT01673932). This study was designed to assess the safety and possible efficacy of hUCB-MC treatment of chronic ischemic stroke. The study is still recruiting participants and is scheduled to transplant 10–40 million viable hUCB-MCs into brain tissue adjacent to the infracted site.

In 2013, two phase I clinical trials on the use of allogeneic hUCB therapy for patients with stroke started almost simultaneously in Taiwan and Korea (ClinicalTrials.gov Identifiers: NCT01438593; NCT01884155). The results of these clinical trials are not yet known, but they are of great interest in connection with the inter-cerebral implantation of allogenic CD34^+^ stem cells (in the first study) and the allogeneic hUCB therapy to treat stroke in patients of all age groups (in the second study). In 2015, three clinical trials on the use of hUCB cells were registered and approved, two of them in USA and one in Taiwan. The purpose of the Taiwanese phase I/II clinical trial is to determine if hUCB intravenous infusion for perinatal arterial ischemic stroke is safe; if late functional outcome is improved; if hUCB treatment improves physiologic response in the child's SSEP & EEG and finally describe the effect of hUCB infusion in altering anatomic findings on MRI (ClinicalTrials.gov Identifier: NCT02460484). The two USA clinical trials using hUCB cells by intravenous injection in acute ischemic stroke patients (ClinicalTrials.gov Identifiers: NCT02433509; NCT02397018) have similar goals but different approaches to the dose and type of the transplanted hUCB cells (in the first study, only the mononuclear fraction is used) and also to the time of their injection in the acute phase of stroke (injection within 72 h and 3–10 days of the stroke, respectively). More recently, in January 2017, a phase II clinical trial was approved (ClinicalTrials.gov Identifier: NCT03004976). The primary objective of this study is to determine the efficacy of a single intravenous infusion of unrelated donor hUCB in improving functional outcomes in patients with ischemic stroke. The large number of clinical trials started in recent years is clear evidence not only of the high interest in hUCB as a source of stem and progenitor cells for the treatment of stroke, but also the success of the preclinical studies.

## Current perspectives in the treatment of peripheral nerve injury

Cell therapy using hUCB is a promising strategy for treatment of peripheral nerve injury (PNI). Nevertheless, currently limited experimental data is available on using hUCB cells for PNI treatment. Wang et al. reported that tissue engineered nerves from hUCB-MSCs-derived Schwann-like cells can effectively repair large defects of the sciatic nerve (Wang et al., 2015). Injection of hUCB-MSCs into the crush-injured segment of the sciatic nerve promotes the functional recovery (Sung et al., 2012). Most cell therapy studies for treating PNI utilize different sources of MSCs (Masgutov et al., 2015, 2016). It was shown, that MSCs from adipose tissue promote neuronal survival in the spinal ganglion, axonal repair and stimulate the regeneration of peripheral nerves. This limited interest in hUCB as source of cells for PNI treatment is probably due to several reasons. First of all, most of PNI are not life threatening. Even standard microsurgery techniques, such as end-to-end repair or autologous nerve transplantation, result in satisfactory recovery rate in most of cases. Secondly, most of complex surgeries for PNI treatment are planned so there is sufficient time to isolate and, if necessary, expand cells for transplantation. Finally, autologous cell therapy is always preferred for treating traumatic injuries. All this limits the appeal of hUCB as source of cells for treating PNI. Still, more research is needed to assess the efficiency of different sources of cells, including hUCB, for PNI treatment.

## Application of genetically modified hUCB cells in neuroregeneration

Despite the importance of further research on hUCB cell transplantation for the treatment of neurodegenerative processes of multiple etiologies, it has become apparent that it is possible to enhance their therapeutic effects, ultimately leading to a significant improvement of both structure and function. For this reason, genetically modified hUCB cells with enhanced expression of neurotrophic and angiogenic factors have attracted the attention of researchers as a means to stimulate post-traumatic regeneration of the spinal cord. Investigations in this direction using hUCB cells to deliver therapeutic genes began relatively recently.

Earlier, using a rat model of spinal cord contusion, we showed that intraspinal transplantation by Hamilton syringe of hUCB-MCs transduced (genetically modified) with adenoviral vector expressing GDNF (hUCB-MCs+Ad5GDNF) improves the motor function and results in a better preservation of tissues at the injury site. This included increasing the number of intact myelinated fibers, compared to transplantation of native hUCB-MCs (Mukhamedshina et al., 2016b). Retro-orbital injections of the construct hUCB-MCs+Ad5GDNF have been shown to stimulate hippocampal neurogenesis in mouse models of Alzheimer's disease (Petukhova et al., 2015). Also, Ikeda et al. demonstrated that it is possible to increase the capillary density in the ischemic area, enhance blood flow and promote the formation of new blood vessels after transplantation of hUCB-MCs expressing vascular endothelial growth factor (VEGF) (Ikeda et al., 2004). Recently, Hei et al. using a rat sciatic nerve crush injury model showed that both hUCB-MSCs and hUCB-MSCs+AdBDNF can improve rat sciatic nerve regeneration, with hUCB-MSCs+AdBDNF showing a greater effect than hUCB-MSCs (Hei et al., 2017).

**Figure 1:**
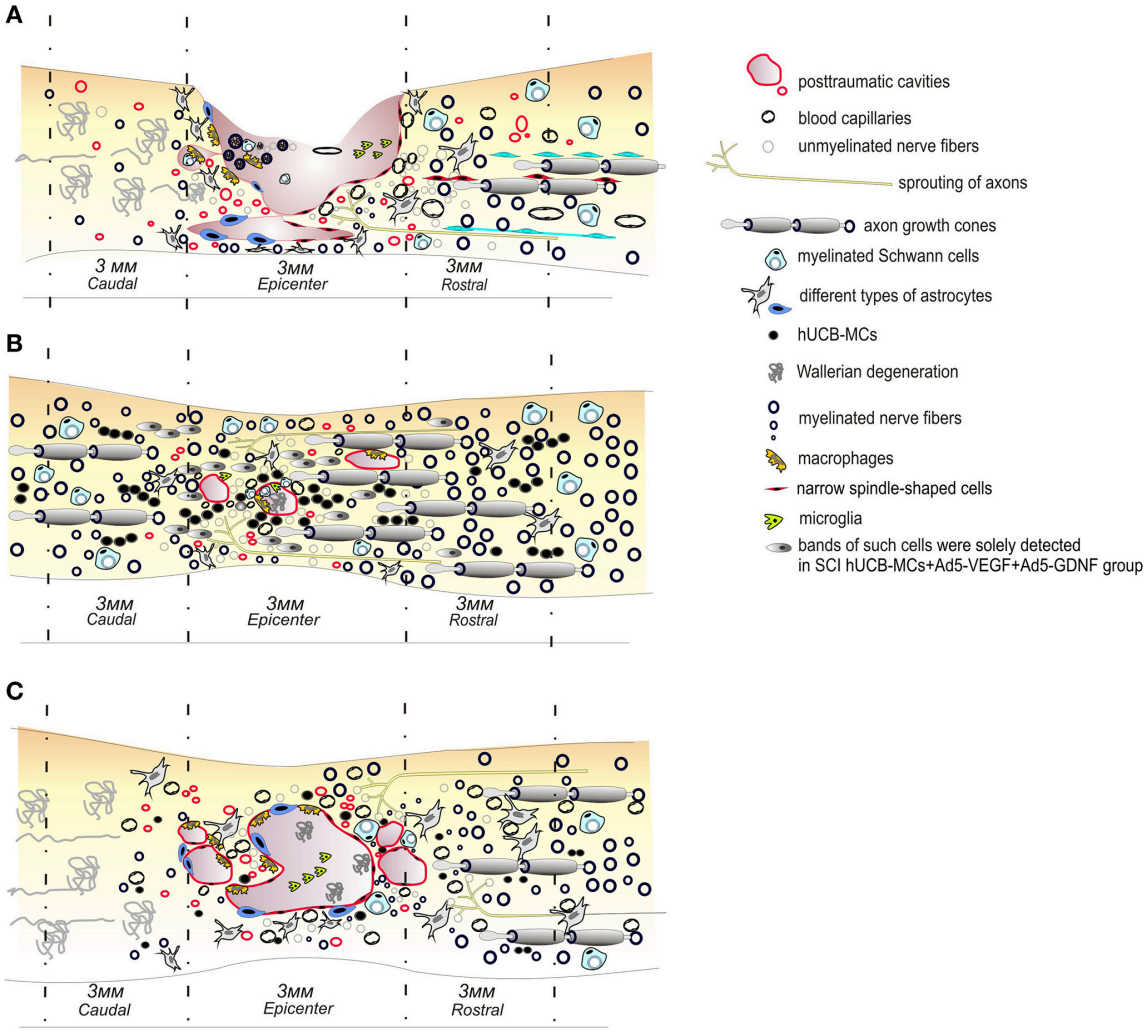
Schematic illustration of pathological and cellular reactions after contusive spinal cord injury in rats **(A)** and transplantation of native **(C)** or genetically modified *vegf* and *gdnf* genes hUCB MCs **(B)**. The transplantation of genetically modified *vegf* and *gdnf* genes hUCB cells leads to a significant improvement in recovery of the motor function, provides for a better preservation of tissues, reduces glial scar formation and also induces prominent axonal sparing/regeneration in comparison to controls or native hUCB MC therapy.

Researchers are beginning to use hUCB cells to deliver two or more therapeutic genes, allowing them to study the possibility of potentiating their neuroregeneration ability. We reported on experiments transplanting hUCB-MCs transduced with adenoviral vectors expressing VEGF and GDNF genes into a SCI site. We showed that this administration leads to a significant improvement in recovery of motor function, provides for better tissue preservation, reduces glial scar formation and also induces prominent axonal sparing/regeneration in comparison to native hUCB-MCs (Figure 1; Mukhamedshina et al., 2016a). A similar construct was tested in a mouse model of ALS, achieving significant symptomatic control and a prolonged lifespan in ALS mice (Robertovich Islamov et al., 2015). Genetically modified hUCB-MCs expressing VEGF and GNDF facilitated the targeted delivery of these factors and extended the survival of motor neurons. The neuroprotective effect was supplemented because these cells also differentiate into glial cells (Rizvanov et al., 2011).

Thus, the use of genetically modified hUCB cells, with enhanced expression of therapeutic genes has started to be intensively explored as a means to increase the efficiency of post-traumatic regeneration. The transplantation of hUCB cells transfected with viral or plasmid vectors encoding genes of neurotrophic or angiogenic factors appears to be more effective than the transplantation of native cells in the treatment of spinal cord injury and neurodegenerative diseases, such as AD and ALS. However, these pre-clinical observations are not yet sufficient to progress into clinical practice. Thus, clinical studies on the use of genetically modified hUCB cells for the treatment of spinal injury, ALS and AD have not yet commenced.

## Conclusions

Human umbilical cord blood (hUCB) is an excellent source of stem and progenitor cells able to exert a neuroprotective effect in neurodegenerative processes. A considerable number of studies have been devoted to the investigation of hUCB cell transplantation in the treatment of traumatic and ischemic injuries of the spinal cord, as well as neurodegenerative diseases. These studies have proven that the accumulated experience has good prospects to be translated into clinical practice. Thus, international clinical trials on the effectiveness of hUCB cells for the treatment of these diseases and injuries are currently being conducted. They will determine the further development of therapies using hUCB cells. It is possible that genetic modification of hUCB cells with therapeutic genes will potentiate their neuroprotective/neuroregenerative activity.

## Author contributions

LG-collection of data on the characteristics of cells derived from umbilical cord blood, compilation these data. YM-collection of data on the transplantation umbilical cord blood-derived cells into the area of spinal cord injury, compilation these data. SA-collection of data on the transplantation umbilical cord blood-derived cells in neurodegeneration deseases, drawing Figure 1. AR-compilation of article content, writing the some chapters.

### Conflict of interest statement

The authors declare that the research was conducted in the absence of any commercial or financial relationships that could be construed as a potential conflict of interest.
